# Pelvic Floor Muscle Training for Urinary Incontinence with or without Biofeedback or Electrostimulation in Women: A Systematic Review

**DOI:** 10.3390/ijerph19052789

**Published:** 2022-02-27

**Authors:** Souhail Alouini, Sejla Memic, Annabelle Couillandre

**Affiliations:** 1 Center Hospitalier Regional d’Orléans, Departement of Gynecologic Surgery, 14 Avenue de L’hôpital, 45100 Orleans, France; 2 EUKCVL, Université d’Orléans, 45100 Orleans, France; sejla.memic@univ-orleans.fr (S.M.); annabelle.couillandre@univ-orleans.fr (A.C.); 3 Laboratoire CIAMS, Université Paris—Sud, EA 4532, 91400 Orsay, France; 4 Laboratoire en Neurosciences, Physiologie et Psychologie, LINP2, Université Paris Nanterre, 92001 Nanterre, France

**Keywords:** pelvic floor muscle training, urinary incontinence, non-pregnant, biofeedback, electrostimulation, extracorporeal magnetic innervation, vaginal cones, whole body vibration training, stress urinary incontinence, mixed urinary incontinence

## Abstract

To determine the effectiveness of pelvic floor muscle training (PFMT) with or without biofeedback or electrostimulation in reducing urinary incontinence and pelvic floor muscle con-traction in non-pregnant women with urinary incontinence. Methods: The following electronic databases were searched: PubMed, Cochrane Central, ClinicalTrials.gov, EU Clinical Trials Register, and sources from NICE, FDA, EMA, and SMC (articles only in English, 2000–2021). Search terms were: urinary incontinence, pelvic floor muscle training or exercises, biofeedback, electrostimulation. We used the PRISMA statement (Preferred Reporting Items for Systematic Reviews and Meta-Analyses) for this systematic review. Relevant articles were selected, data were extracted, and quality was assessed. Data were extracted in predesigned form, followed by narrative synthesis. Results: Following the search, 15 RCTs were retrieved using the strict inclusion and exclusion criteria, assessing 2441 non-pregnant women with urinary incontinence. Of the 15 studies, 7 were low risk, 5 were medium risk, and 3 were high-risk studies. Of the 2441 patients, 970 were in PFMT, 69 were in extracorporeal magnetic innervation (ExMi) or with PFMT + BF, 30 were in electrostimulation (ES), 21 were in whole body vibration training (WBVT), 23 were in pelvic floor muscle + abdominal muscle therapy (PFM + AMT), 326 were in PFMT + biofeedback, 93 were in vaginal cones (VC), 362 were in PFMT + education, 318 were in education, and 229 were in control groups. The most often measures employed were pad tests, bladder diary, and questionnaire on the quality of life. Stress, urge and mixed urinary incontinence were studied. In all RCT, PFMT significantly reduced urinary incontinence, essentially SIU and MUI, when compared with the control group before and after treatment. Overall, out of 997 PFMT or PFMT + education patients, 504 patients (50.5%) showed improvement in urinary incontinence, and 218 became continent (21.8%) (negative pad test). In total, 62% of patients significantly reduced their urinary incontinence or cured it and improved their pelvic floor muscle contraction. All other physiotherapist techniques also significantly reduced urinary leakages, e.g., vaginal cones, biofeedback, ExMI, and WBVT when compared with the control group. There were no significant differences between these methods in reducing the severity of urinary incontinence. Conclusion: PFMT alone or with bio-feedback or electrostimulation was effective in reducing urinary incontinence and improving pelvic floor muscle contraction. PFMT when compared with other interventions such as bio-feedback, VC, and WBVT did not show significant differences but was superior to the control group. RCT studies with similar parameters used for measuring the outcomes need to be included.

## 1. Introduction

Urinary incontinence (UI) is defined as involuntary leakage of urine through the urethra by the International Continence Society (ICS) [1]. It is considered to be a health, social, and hygienic concern. UI affects 6–10% of the population [1]. UI maybe divided into three subtypes: stress urinary incontinence (SUI), urge urinary incontinence (UUI), and mixed urinary incontinence (MUI) based on behavioral symptoms and pathophysiology [1,2]. Stress urinary incontinence (SIU) is the common type of UI among women. SUI occurs during efforts such as sneezing, coughing, or exertion. It is found in either pure or mixed form in one-third of women [3]. SUI is caused due to a lack of strength in the urethral sphincter muscles, pelvic floor muscles, connective tissues, and fasciae [4].

Urge incontinence is defined by urinary leakage following a sudden and strong desire to urinate [3]. The combination of SIU and UUI is referred to as mixed urinary incontinence. Around 25–45% of all women suffer from involuntary urine loss at some stage in their lives, thus making UI one of the most frequent complaints in this population [5]. Non-pregnant or prepartum women were less likely to suffer from UI compared with postpartum women. However, in the last few decades, there has been an increase in the number of women experiencing incontinence among non-pregnant or prepartum women. Currently, the importance of prevention and treatment of UI has grown. Hence, increased attention has been given to conservative treatments for UI.

Conservative management of UI has been recognized as the first-line management, which includes physical therapies, behavior modification, and pharmacological intervention, specifically for SUI [2]. Some examples of conservative treatments that are used in the management of UI are physical therapies and pelvic floor muscle training (PFMT) alone or combined with other treatments—for example, electrical stimulation, biofeedback, and vaginal cones. These are the standard management procedures [3]. Pelvic floor muscle training (PFMT) is considered as the first-line treatment, as suggested by the International Continence Society (ICS), and it has been proven to guarantee results for UI. Guided PFMT also improves adherence positively and promotes self-efficacy behavior among the participants [4]. PFMT provides support to the pelvic organs and helps in the closure of the urethral sphincter muscles, thus resulting in improvement in incontinence. Hence, it is prescribed for increasing strength, endurance, and muscle coordination [6,7]. Previously, many authors have performed various reviews regarding the implications, causes, and treatments for UI, but no systematic review has been performed in measuring the efficacy of PFMT among non-pregnant women.

This review focused on analyzing the efficacy of pelvic floor muscle training in the treatment of UI and its effect on the improvement in muscle strength and endurance among non-pregnant women.

## 2. Material and Method

This systematic review was carried out according to the pre-specified PRISMA protocol that was implemented before initiation of the study. The protocol was followed throughout the process of study selection, data extraction, quality assessment, and data synthesis.

### 2.1. Search Strategy

The following databases (PubMed, Cochrane Central, ClinicalTrials.gov, EU Clinical Trials Register), grey literature sources (NICE, FDA, EMA, SMC), and snowballing search were conducted by using Boolean operators and limiting the search strategy to only articles published in English from the 1 January 2000 to the 1 March 2021. The search strategy used was as follows: (pelvic floor muscle therapy) OR (pelvic floor muscle physiotherapy) OR (pelvic floor muscle exercise) OR (pelvic muscle physiotherapy) OR (pelvic muscle therapy) OR (pelvic muscle exercise) OR (pelvic muscle physical therapy) OR (pelvic floor muscle physical therapy) OR (pelvic floor muscle training) OR (PFMT) OR (pelvic muscle training) OR (pelvic floor muscle electrostimulation) OR (pelvic muscle electrostimulation) OR (pelvic floor muscle electrical stimulation) OR (pelvic muscle electrical stimulation) OR (pelvic floor muscle training) OR (PFMT) OR (pelvic muscle training) OR (pelvic floor muscle electrostimulation) OR (pelvic muscle electrostimulation) OR (pelvic floor muscle electrical stimulation) OR (“pelvic floor training” OR “pelvic floor muscle therapy” OR “PFMT”) AND (urinary incontinence) OR AND (women OR female). The equivalent search keywords or the syntonic terms were used in other databases.

### 2.2. Study Selection and Criteria

Selection of studies was based on predesigned inclusion and exclusion criteria following the searches in relevant databases. Initially studies were identified based on screening of title and abstract, followed by full-text screening and including the final studies. Screening of the articles was conducted by two reviewers. The inclusion and exclusion criteria were based on the PICOS format (Table 1). The population criteria included only non-pregnant women patients suffering from UI. Post-surgical patients were excluded from the study. Intervention criteria included pelvic floor muscle training with or without biofeedback therapy with the exclusion of pharmacological interventions. The outcome of our interest included muscle strength, the endurance of PFM, UI and urinary leakage, and exclusion of another outcome. RCT studies were included, while the rest of all other types of experimental or analytical studies were excluded. A limitation was placed by including only English language studies published from the 1 January 2000 to 31 March 2021.

### 2.3. Data Extraction, Quality Assessment, Data Synthesis

Data extraction was performed in Microsoft Excel after reviewing all the final included studies. The data that were extracted from all the studies were the studies’ first author, year of publication, study design, inclusion criteria, intervention groups sample size, drop out, study duration, study outcome. During data extraction, no authors were contacted. Quality assessment of the studies was carried out using, “The Cochrane risk of bias 2 tool”. The quality of the studies was based on the following questions: randomization, deviation from interventions, missing outcome reporting, measuring the outcomes, and selective reporting of outcomes. Based on the following questions, studies were marked as low, medium, or high risk of bias. Following the data extraction, narrative synthesis was performed. Heterogeneity tests and meta-analysis of the studies were not performed.

## 3. Results

### Study Selection

**Figure 1:**
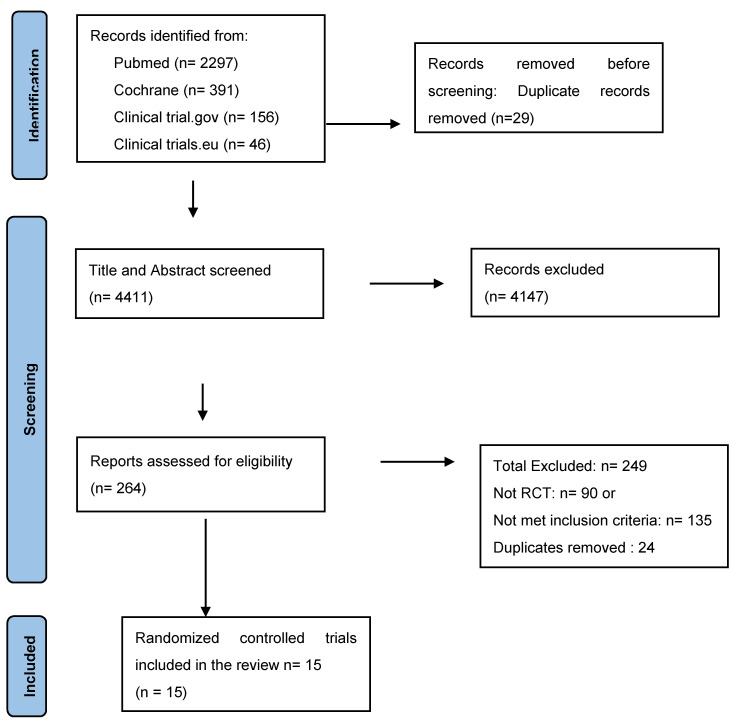
Study selection, flow diagram.

After systematic retrieval, 264 articles were selected from 4411 citations after duplication removal, proper title, and abstract screening. Following this, full-text screening of 264 articles was performed, out of which 15 were finally included, as shown in Figure 1. Finally, 15 articles consisting of 2441 non-pregnant women who had UI were selected for analysis.

## 4. Narrative Synthesis

### 4.1. Study and Population Characteristics

In terms of study and participants characteristics, there was a lot of homogeneity across all the studies [1,2,3,4,5,6,7,8,9,10,11,12,13,14,15,16], as shown in Table 2. Among the 15 RCTs selected, 3 were performed in Brazil [3,5,8], 3 were performed in Canada [10,13,15], and 2 in Iran [7,9] while the rest were performed in Poland [1], UK [11], Sweden [4], Bangladesh [14], Norway [12], Hong Kong [2], and Turkey [16].

Patients randomized across all the studies were 2441, of which dropout was 227, and 2214 patients completed the studies. The lowest drop-out rate was 2 in Dumoulin et al. [10], and the highest was in Wagg et al. [14]

Intervention groups across all the studies were PFMT, extracorporeal magnetic innervation (ExMi), whole body vibration training (WBVT), pelvic floor muscle + abdominal muscle therapy (PFM + AMT), PFMT + biofeedback, pelvic floor muscle therapy + electrical stimulation (PFMT + ES), vaginal cones (VC), PFMT + education, education, and control. Out of the 15 studies, 11 studies [2,4,7,9,10,11,13,15,16] comprised two intervention groups, while 3 studies [1,5,10] comprised three interventions groups, and 1 study [3] comprised 4 intervention groups. The total number of patients in the PFMT group was 970 [1,2,5,7,13,17,18], ExMi was 69 [1,16], WBVT was 21, PFM + AMT was 23, PFMT + biofeedback was 326 [11,18], VC was 93 [3,8], PFMT+ education was 318 [14], education was 290 [14], and control was 229 [1,3,4,5,7,10,12,13].

The most important inclusion criterion across all the studies was aged above 18 years suffering from UI. Pregnant women or women in the early postpartum period (before 10 weeks after delivery) were excluded from the study. Study duration among all the studies ranged from 4 weeks to 104 weeks. A total of 3 studies [3,4,12] had a study duration of 26 weeks, 4 studies [2,8,13,17] had a duration of 12 weeks, and 3 studies lasted 8 weeks [7,10,18]. The remaining 5 studies had a duration of 52 weeks [5], 24 weeks [14], 104 weeks [11], 13 weeks [9], and 4 weeks [1]. The most common outcomes collected across the 15 studies were UI, PFM strength, endurance, urinary leakage, QoL, symptoms, PFM activity or function, vaginal squeeze pressure, self-esteem, and urodynamic test.

### 4.2. Overall Quality and Risk of Bias Assessing of Studies

**Figure 2:**
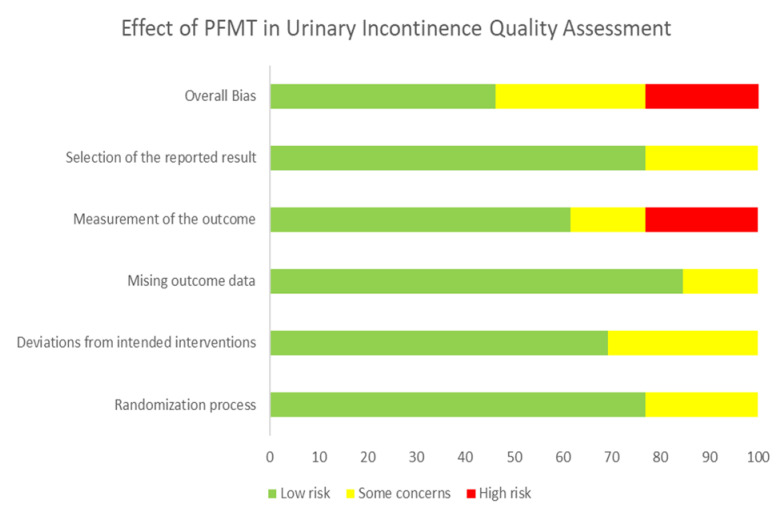
Risk of bias for studies as percentage.

**Figure 3:**
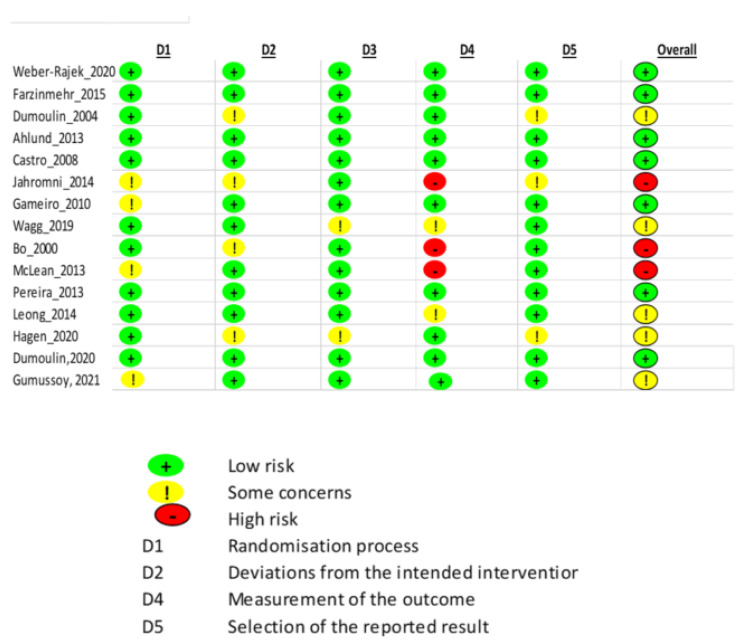
Cochrane risk of bias for each component across all the studies.

Quality assessment of the studies was conducted using the “Cochrane ROB2” tool, as shown in Figure 2 and Figure 3 and Table 3. The studies were classified as at low risk of bias, some concerns, or high risk of bias. Overall, 7 studies [1,3,4,5,8,9,17] reported a low risk of bias, 3 studies [12,13] reported a high risk of bias, while the remaining 5 studies reported medium [2,10,11,14,18], or some risk of bias, as represented in Figure 3. Almost half of the studies had a low risk of bias in terms of the randomization process, missing outcome data, and selection of reported result, as shown in Figure 2. Around 76.9%, 69.2%, 84.6%, 61.5%, and 76.9% across all the studies reported low risk of bias in terms of the randomization process, deviation of intended interventions, missing outcome data, measurement of the outcome, and selection of the reported result, respectively. Only 20% across all the studies reported a high risk of bias in terms of measurement of the outcome.

### 4.3. Study Outcome

Regarding outcome reporting, there was a lot of heterogeneity among the studies in terms of scales used for measuring the outcomes, reporting age, and other outcomes, as shown in Table 2. The most used scales for measuring muscle contraction, incontinence, and leakage were the pad test, VAS, I-QOL, Perineometer, ICIQ-UI SF, IIQ, and B-FLUTS. The age of women with incontinence ranged from 29.4 to 85 years across all the studies. In terms of reporting the outcome, there was a lot of dissimilarity across the studies.

Outcome on muscle contraction or muscle strength was reported by eight studies [3,5,8,11,16], urinary incontinence was reported by all the studies, and endurance was reported by one study [4].

In terms of muscle contraction, four studies [3,5,9,16] showed significant improvement between the groups; however, the instruments used for measuring the outcome for muscle contraction were different in all these studies. In four studies, improvements in muscle contraction were observed at the end of the study from baseline. In Hagen et al. [11], 8.5% of the patients in the PFMT + biofeedback and 6% in the PFMT group reported improvement in muscle contraction at 6 months. Ahlund et al. [4] showed a significant increase in PFM contraction from baseline in both groups at 26 weeks. Gameiro et al. [8] showed significant improvement from baseline in both the groups at 6 months but not at 12 months. For Gumussoy et al. [16], PFMC increased in both groups (PFMT with and without ExMI). However, there are few studies on ExMi and WBVT, and other studies are necessary to evaluate the use of these methods.

On the other hand, there were no significant results found for the endurance outcome across the studies. However, in one study by Ahlund et al. [4] at 26 weeks, improvement in endurance was observed from baseline.

All RCT studies showed significant reduction in severity of urinary incontinence from baseline after PFMT. However, the scale used for measuring the outcomes varied across the studies. For Dumoulin et al. [15], 70% of patients in individual PFMT group and 74% in group-based PFMT had a significant reduction in urinary incontinence at one year. For Gumussoy et al. [16], PFMT with BF with and without ExMI allowed the reduction in urinary loss. In Hagen et al. [11], 60% of patients in the PFMT + biofeedback and 62.6% in the PFMT group reported an improvement in symptoms at 24 months. In Ahlund et al. [4], a significant improvement was observed in both PFMT and control group at 26 weeks. In Jahromi et al. [7], a significant improvement from baseline was observed in both PFMT and control group for incontinence quality of life at 8.5 weeks, and in Leong et al. [2], more than 90% reduction was observed in PFMT group compared with control (7.2%) at 12 weeks.

Eight studies [2,3,5,7,10,14,15,16], used a similar outcome, i.e., the pad test method for measuring the outcome of urinary leakage, while the remaining three studies used different methods. In four studies, individual improvement was observed in the intervention groups from baseline. In Castro et al. [3], improvement in urinary leakage using the pad test was observed in 46% in PFMT, 48% in ES, 46% in VC, and 8% in the control at 26 weeks. In Jahromi et al. [7], a significant improvement from baseline was observed in both PFMT and control group for frequency of urine leakage and amount of urine leakage at 8.5 weeks. In Gameiro et al. [8], a significant improvement was observed from baseline in both the groups at 6 months but not at 12 months. Pereira et al. [5] reported significant decrease in urinary leakage in PFMT and VC group from baseline as compared with the control group at 52 weeks.

### 4.4. Sensitivity and Subgroup Synthesis

Overall, out of 997 PFMT or PFMT + education patients, 504 patients (50.5%) showed improvement in urinary incontinence, and 218 became continent (21.8%) (negative pad test). In total, 62% of patients significantly reduced their urinary incontinence or cured it and improved their pelvic floor muscle contraction.

On the other hand, significant improvements in endurance, muscle contraction, and urinary leakage were observed in 49 patients (23%), 168 patients (79.2%), and 97 patients (46%), respectively. However, the results across the outcome cannot be compared between the intervention groups, as the scales used for measuring the outcome varied across studies, except for urinary leakage.

## 5. Discussion

This systematic review demonstrated that PFMT is effective in reducing UI and improving muscle contraction.

Although previously, many systematic reviews have been published that assessed the efficacy of PFMT on pregnant and non-pregnant women, this review specifically focused on non-pregnant women [17,18,19,20,21]. Apart from UI, this is the first review that also focused on PMFT efficacy on endurance, muscle contraction, and urinary leakage. Moreover, in this review, only RCT studies were included, followed by quality assessment using the Cochrane risk of bias tool.

It focused on finding the clinical effectiveness of PFMT on non-pregnant women suffering from UI. Indeed, PFM dysfunction is associated with UI [22,23,24,25].

Overall, PFMT has almost proven to be effective in all the studies conducted. Some have used PFMT with biofeedback, which also has shown efficacious result [12,26]. It improved not only the physical but also the psychosocial aspects of women. Significant decline in depressive symptoms, with an improvement in UI severity and quality of life, was observed following the treatment [1]. There was also a significant decrease in incontinence among the groups treated with PFMT. Other observational studies also found a significant improvement in urinary continence after PFMT [26,27,28,29,30]. In almost every study, by increasing the pelvic muscle strength in women, it improved the quality of life index as well. Hence, it could be hypothesized that PFMT is a successful method for the treatment of incontinence and is recommended as first-line treatment [31].

Our study demonstrated that PFMT was effective in reducing UI. The duration of analyzed studies varied between 4 weeks and 52 weeks. A long-term follow-up was not evaluated, as our study was not focused on the recurrence of UI. The question of the duration of benefit effects of PFMT is important. However, as it is a conservative and minimally invasive treatment, other PFMT could be prescribed in case of recurrence of UI.

Another significant finding is that most of the studies, which used interventions such as ES, VC, WBVT, ExMi innervation, etc. along with PFMT, concluded that all these interventions were proven to be equally effective in treating UI. All these interventions also helped in the significant decrease in incontinence and improvement in quality of life [32,33,34]. No such significant difference was seen among the interventions [1,2,3,5,9]. In this review, in terms of study characteristics, the studies were similar, and in terms of reporting of outcomes, the studies were dissimilar. After conducting the sensitivity synthesis by vote-counting of the studies, all the good quality studies either showed significant improvement from baseline or significant improvement between the intervention groups. The majority of the patients had improvement in UI and in urinary leakage. However, improvement in muscle contraction and endurance could not be well established, as the scales used were different across the studies, but the pad test was used for measuring urinary leakage across the good-quality studies [3,5,8].

### Strengths and Limitations of the Study

This study also has some important strengths and weaknesses. First, the search strategy used in this review was very robust, as it looked for citations on five databases and from four international grey literature sources (NICE, EMA, FDA, SMC). Throughout the review, the screening of the articles was based on the predesigned protocol, which was unchanged throughout the study. The quality of the studies was assessed and was taken into consideration while assessing the efficacy. No authors were contacted for gathering the missing data. Our systematic review was limited to articles written in English, which could constitute some bias. However, the majority of RCTs are written in English to reach an international scientific audience. We limited our study to the period 2000–2021; as methods and machine of physiotherapy changed, we did not extend our review before the year 2000. Meta-analysis was also not possible, as the scales used for measuring the outcomes and the measures of parameters were different across the studies.

However, better-quality RCT studies need to be included with similar parameters used for measuring the outcomes. Thus, this narrative sensitive synthesis showed enough evidence about the efficacy of PFMT in reducing UI and improving pelvic floor muscle contraction. Therefore, PFMT should be proposed before invasive surgical treatment for management of urinary incontinence.

## Figures and Tables

**Table 1:** Inclusion and exclusion criteria of the study.

Criteria	Inclusion	Exclusion
Population	Women/Female Mixed population Pre- and post-menopause women Postpartum women	Post-surgical patients Pregnant women Animal Male
Intervention	Physiotherapy Pelvic floor muscle training (PFMT) PFMT with electrostimulation	Other interventions Pharmacological interventions
Comparator	Any comparator Placebo	Pharmacological PFMT with pharmacological Pharmacological with electrostimulation
Outcome	Muscle strength Endurance Urinary incontinence Urinary leakage	Any other clinical or biochemical outcome
Study	RCT	Analytical study Non-randomized study Qualitative study Narrative review Laboratory study
Language	English	Non-English
Year	2000–Present	Before 2000

**Table 2:** Study and population characteristic.

Study Name Authors	Country	Study Type	Inclusion Criteria	Sample Randomized	Intervention Groups (Sample Size)	Duration of Study	Study Outcome	Drop out
Gumussoy et al. [16] 20	Turkey	RCT	Women with SIU.	51	26 EMG-BF 25 EMG-BF + ExMI.	8 weeks	1 h pad test (grams of urine loss) 3-day bladder diary I Qol pelvic floor muscle contraction force measured via a perineometer and Modified Oxford Scale (MOS).	23
Dumoulin et al. [15] 2020	Canada	RCT	Stress or mixed UI in older women.	362	Group PFMT (178) vs. individual PFMT (184)	12 weeks 1-year follow-up	%reduction in UI episodes in 1 year I Qol	43
Weber-Razek et al. [1] 2020 [1]	Poland	RCT	Women patients with urinary incontinence.	128	PFMT (44) ExMI (44) Control (40)	4 weeks	Urinary incontinence, severity, QoL	17
Farzinmehr et al. [9] 2015	Iran	RCT	Women patients with 4.5-year history of urinary incontinence.	46	WBVT (24) PFMT (22)	3 months (13 weeks)	Urinary incontinence, severity, PFM strength	3
Chantale et al. 2004 [10]	Canada	RCT	Women patients exhibiting symptoms of stress urinary incontinence at least once per week for 3 months or more after their last delivery.	64	PFMT (21) PFM + AMT (23) Control (20)	8 weeks	PFM function, muscle strength, endurance, rapidity of contraction, urine leakage incontinence	2
Hagen et al. [11] 2020	UK	RCT	Women patients aged 18 years or older and newly presenting with clinically diagnosed stress or mixed urinary incontinence and urine leakage.	600	PFMT + Biofeedback (300) PFMT (300)	24 months (104 weeks)	Incontinence, severity, symptoms, QoL, endurance, PFM strength	7
Ahlund et al. [4]. 2013.	Sweden	RCT	Women patients having urinary incontinence after 10–16 weeks postpartum.	98	PFMT (49) Control (49)	6 months (26 weeks)	PFM strength, endurance, incontinence, symptoms, vaginal squeeze pressure	16
Castro et al. [3] 2008	Brazil	RCT	Women patients having had urodynamic stress incontinence of at least 3 stress incontinence episodes in a week.	118	PFMT (31) ES (30) VC (27) Control (30)	6 months (26 weeks)	QoL, urine leakage, urodynamic test	17
Jahromi et al. 2013 [7]	Iran	RCT	Women having Quid score for incontinence type (stress score ≥ 4, clinical symptoms of urinary incontinence within the last 6 months).	50	PFMT (25) Control (25)	2 months (8.5 weeks)	Urinary incontinency, urine leakage, self-esteem, QoL, self-esteem	2
Gameiro et al. [8] 2010	Brazil	RCT	Women patients having symptoms of SUI and urge incontinence.	103	VC (51) PFMT (51)	12 weeks	Urinary leakage, PFM contraction	0
Wagg et al. [14] 2019	Bangladesh	RCT	Women patients having current urinary incontinence, with a positive response of urinary leakage with urgency, stress, or drops of urine loss.	625	PFMT + Education (335) Education (290)	24 weeks	Urinary leakage	46
Bo et al. [12] 2000	Norway	RCT	Women patients having history of stress urinary incontinence and >4 g of urine leakage.	59	PFMT (29) Control (30)	6 months (26 weeks)	Incontinence, symptoms, QOL	6
McLean et al. [13] 2013	Canada	RCT	Women patients having symptoms of SUI with or without urge incontinence.	40	PFMT (20) Control (20)	12 weeks	Incontinence, urinary flow	5
Pereira et al. [5] 2013	Brazil	RCT	12-month post-menopausal women patients having at least one episode of SUI symptom.	45	PFMT (15) VC (15) Control (15)	12 months (52 weeks)	Urinary leakage, PFM strength, QoL	4
Leong et al. [2] 2015	Hong Kong	RCT	Women patients having a clinical diagnosis of SUI, UUI, or MUI.	55	PFMT + BT + Education (27) Education (28)	12 weeks	QoL incontinence episodes	0

(ExMi, extracorporeal magnetic innervation; WBVT, whole body vibration training; PFM + AMT, pelvic floor muscle + abdominal muscle therapy; PFMT, pelvic floor muscle therapy; ES, electrical stimulation; VC, vaginal cones).

**Table 3:** Study outcome.

Study Name	Scales Used for Measuring Outcomes	Risk of Bias	Age (Years)	Result for Muscle Contraction	Result for Endurance	Result for Urinary Incontinence
Gumussoy et al. [16] 2021	Pad test, 3-day bladder diary I Qol PFMC with a perineometer and Modified Oxford Scale	Some concern.	50.92 years (SD 8.88).	Pelvic floor muscle contraction force significantly increased in both groups.		Both groups achieved reductions in urine loss during treatment. -The rate of decrease in pad test values of the EMG-BF + ExMI group was higher. - Significant differences in the number of urinations on a daily basis (9 vs. 8 for the EMG-BF and 9 vs. 7 in the EMG − BF + ExMI group.
Dumoulin et al. [15] 2020	%reduction in UI episodes in 1 year, 7-day bladder diary Qol	Low risk.	Age, 67.9 [5.8] years			Significant reduction in leakage episode frequency at 12 weeks and 1 year for both groups’ median percentage reduction in urinary incontinence episodes at 1 year of 70% in individual PFMT compared with a 74% reduction in group-based PFMT.
Weber-Razek et al. [1]	RUIS, KHQ	Low risk.	Mean (Range): 68.77 (45 to 78)			Statistical improvement in urinary incontinence severity in PFMT
Farzinmehr et al. [9]	VAS, I-QOL	Low risk.	Range: 36 to 48	WBVT was effective in PFM strength similar to PFMT.		WBVT was effective in reducing the severity of incontinence similar to PFMT. Increasing I-QOL questionnaire score. No significant difference was observed between the WBVT and PFMT groups.
Dumoulin et al. [10]	Pad test, VAS, UDI, IIQ, pelvic floor muscle dynamometer	Some concern.	<45	No significant improvement was observed between the PFMT, PFM + AT, and control group.		Significant improvement was observed in the PFMT and PFM + AT group compared with control.
Hagen et al. [11]	ICIQ-UI SF, PGII	Some concern. No objective measure of urinary leakage.	Mean (SD) PFMT + Biofeedback: 48.2(11.6) PFMT: 47.3(11.4)	No significant difference was found between the PFMT + biofeedback (8.5%) and PFMT group (6%) at 6 months.		No clinical or significant difference was observed between PFMT + biofeedback and PFMT groups. 60% in the PFMT + biofeedback and 62.6% in the PFMT group reported improvement in symptoms at 24 months.
Ahlund et al. [4]	BFLUT Symptoms Module ICIQ FLUTS Perineometer OGS	Low risk both groups received instructions on how to contract PFM and, vaginal palpation.	Mean (SD) 33 (3.6)	Muscle strength: No statistically significant difference was observed between PFMT and Control group. However, a significant increase was observed from baseline in both groups.	No significant difference was observed between PFMT and Control groups. However, there was an increase in endurance from baseline in both groups.	Significant improvement was observed in both PFMT and control group from baseline.
Castro et al. [3]	Pad test I-QOL OGS	Low risk.	Mean ± SD PFMT: 56.2 ± 12.5 ES: 55.2 ± 12.8 VC: 52.6 ± 11.2 Control: 52.6 ± 11.2	Significant improvement was observed in the PFMT compared with the ES and VC groups.		Significant decrease in pad weight or improvement in urinary leakage in PFMT, ES, and VC group compared with control. However, no significant difference between ES, VC, and PFMT.
Jahromi et al. [7]	QUID ICIQ Self-esteem questionnaires	High risk.	60–74 years			Significant difference was observed between the PFMT and the control group for frequency of urine leakage.
Gameiro et al. [8]	VAS Pad test Perineometer	Low risk.	Mean VWC: 49 PFMT 48	No statistical difference Was observed between the VWC and PFMT group.		Significant improvement was observed from baseline in both the groups at 6 months but not at 12 months. NS difference between the VWC and PFMT group.
Wagg et al. [14]	EuroQoL Questionnaire EQ5D	Some concern.	Mean (SD) PFMT + Education: 64.5 (4.2) Education: 64.7 (4.1)			A significant decrease in leakage was observed in the PFMT + education group compared with the only education group.
Bo et al. [12]	QoLS-N B-FLUTS	High risk.	Mean (SD) PFMT: 49.6 (10.0) Control: 51.7 (8.8)			Significant improvement in sex-life, social life, and physical activity in PFMT group. NS difference between the groups.
McLean et al. [13]	Pad test UDI-6 IIQ-7 3-day bladder diary	High risk.	Mean ± SD PFMT: 49.5 ± 8.2 Control: 54.0 ± 8.4			Significant improvements in in PFMT group on the impact of SUI compared with the control group. Pad test NS difference between the PFMT group and the control group.
Pereira et al. [5]	Pad test Perina Stim device	Low risk.	Median (min, max) PFMT: 62 (51, 85) VC: 64 (52, 83) Control: 62(51, 80)			Significant decrease in urinary leakage in PFMT and VC group from baseline compared with the control group.
Leong et al. [2]	IIQ-SF-7 UI7	Some concern.	Mean (± SD) 74.3 ± 4.6			Significant reduction in urinary leakage (>90%) in the PFMT + BT + education group compared with the education group (7.2%).

(VAS, Visual Analogue Scale; UI-7, Urinary Incontinence; IIQ-SF-7, Incontinence Impact Questionnaire-Short form; UDI-6, Urogenital Distress Inventory; QoLS-N, Norwegian version of the Quality of Life Scale; B-FLUTS, Bristol Female Lower Urinary Tract Symptoms Module; EQ5D-; ICIQ, International Consultation on Incontinence Questionnaire-urinary incontinence; QUID, Questionnaire for urinary incontinence diagnoses; OGS, Oxford Grading Scale; I-QOL, Incontinence quality of life; RUIS, Revised Urinary Incontinence Scale; KHQ, King’s Health Questionnaire).

## Data Availability

Data are available from the coresponding author under reasonable request.
